# Emerging Therapeutic Strategies in Small-Cell Lung Cancer: From Adaptive Resistance to Precision Therapy

**DOI:** 10.3390/ph19081217

**Published:** 2026-08-02

**Authors:** Jun Kim, Seounghun Kang

**Affiliations:** 1 Department of Chemistry, Soongsil University, Seoul 06978, Republic of Korea; seallingfx@gmail.com; 2 AI-Bio Convergence Research Institute, Soongsil University, Seoul 06978, Republic of Korea

**Keywords:** small-cell lung cancer, adaptive resistance, lineage plasticity, DLL3, immune redirection, antibody–drug conjugate, DNA damage response, biomarker, treatment sequencing, precision oncology

## Abstract

**Background/Objectives**: Small-cell lung cancer (SCLC) remains one of the most therapeutically challenging malignancies despite recent advances in chemoimmunotherapy. Although platinum–etoposide combined with programmed death ligand 1 (PD-L1) blockade has modestly improved survival in extensive-stage disease, most patients ultimately develop treatment resistance. This review examines the evolving biological basis of therapeutic resistance and discusses how emerging treatment strategies may be integrated into biologically informed clinical development. **Methods**: We conducted a narrative review of recent translational studies, clinical trials, regulatory updates, and major oncology meeting presentations addressing emerging therapeutic strategies, biomarkers, and adaptive resistance mechanisms in SCLC. **Results**: Advances in molecular profiling have established that SCLC comprises dynamically evolving transcriptional states characterized by lineage plasticity, replication-stress dependency, immune suppression, and antigen remodeling. These biological insights have accelerated the development of delta-like ligand 3 (DLL3)-directed immune-redirection strategies, next-generation antibody–drug conjugates, DNA damage response-targeting therapies, and epigenetic approaches. Across these platforms, however, clinical translation remains constrained by antigen heterogeneity, biomarker instability, lineage plasticity, immune dysfunction, and cumulative toxicity. We organize the available evidence across three clinically relevant treatment windows—induction, post-induction residual disease, and relapse—to clarify how therapeutic timing may influence the role and expected clinical performance of each strategy. **Conclusions**: Future clinical development should prioritize the prospective validation of dynamic biomarkers and treatment strategies tailored to specific disease stages. Such an approach may improve patient selection, guide therapeutic sequencing, and increase the durability of treatment benefit in extensive-stage SCLC.

## 1. Introduction

Small-cell lung cancer (SCLC) accounts for approximately 13–15% of all lung cancers and is a clinically aggressive neuroendocrine malignancy associated with poor clinical outcomes [1]. SCLC is characterized by rapid cellular proliferation, a high propensity for early metastatic dissemination, and extensive genomic instability [2]. Although many patients initially respond to platinum-based treatment, responses are generally not durable, and disease recurrence or progression is common, particularly in extensive-stage disease [2,3]. Platinum–etoposide chemotherapy has therefore remained the backbone of treatment for several decades.

More recently, the incorporation of immune checkpoint inhibitors into first-line platinum–etoposide chemotherapy modestly improved survival outcomes in extensive-stage SCLC (ES-SCLC), establishing chemoimmunotherapy as the current standard of care [4,5]. Nevertheless, the absolute survival benefit associated with programmed death ligand 1 (PD-L1) blockade remains modest, and durable long-term disease control is uncommon [4,5,6]. SCLC has also historically been difficult to treat with conventional molecularly targeted therapies because recurrent actionable kinase alterations are uncommon and its genomic landscape is dominated by tumor suppressor loss, particularly involving *TP53* and *RB1* [1].

Advances in molecular profiling have demonstrated that SCLC is not a biologically homogeneous disease but comprises transcriptionally and phenotypically distinct states associated with different lineage programs, immune characteristics, and therapeutic vulnerabilities [7,8]. Subtype-defining transcription factors including achaete-scute homolog 1 (*ASCL1*), neurogenic differentiation factor 1 (*NEUROD1*), POU class 2 homeobox 3 (POU2F3), and yes-associated protein 1 (YAP1) have collectively redefined the conceptual framework of SCLC biology and have accelerated the development of subtype-oriented therapeutic approaches [7]. Increasing recognition of lineage plasticity, replication-stress dependency, epigenetic remodeling, and immune suppression has further emphasized the dynamic nature of therapeutic resistance in SCLC.

**Figure 1:**
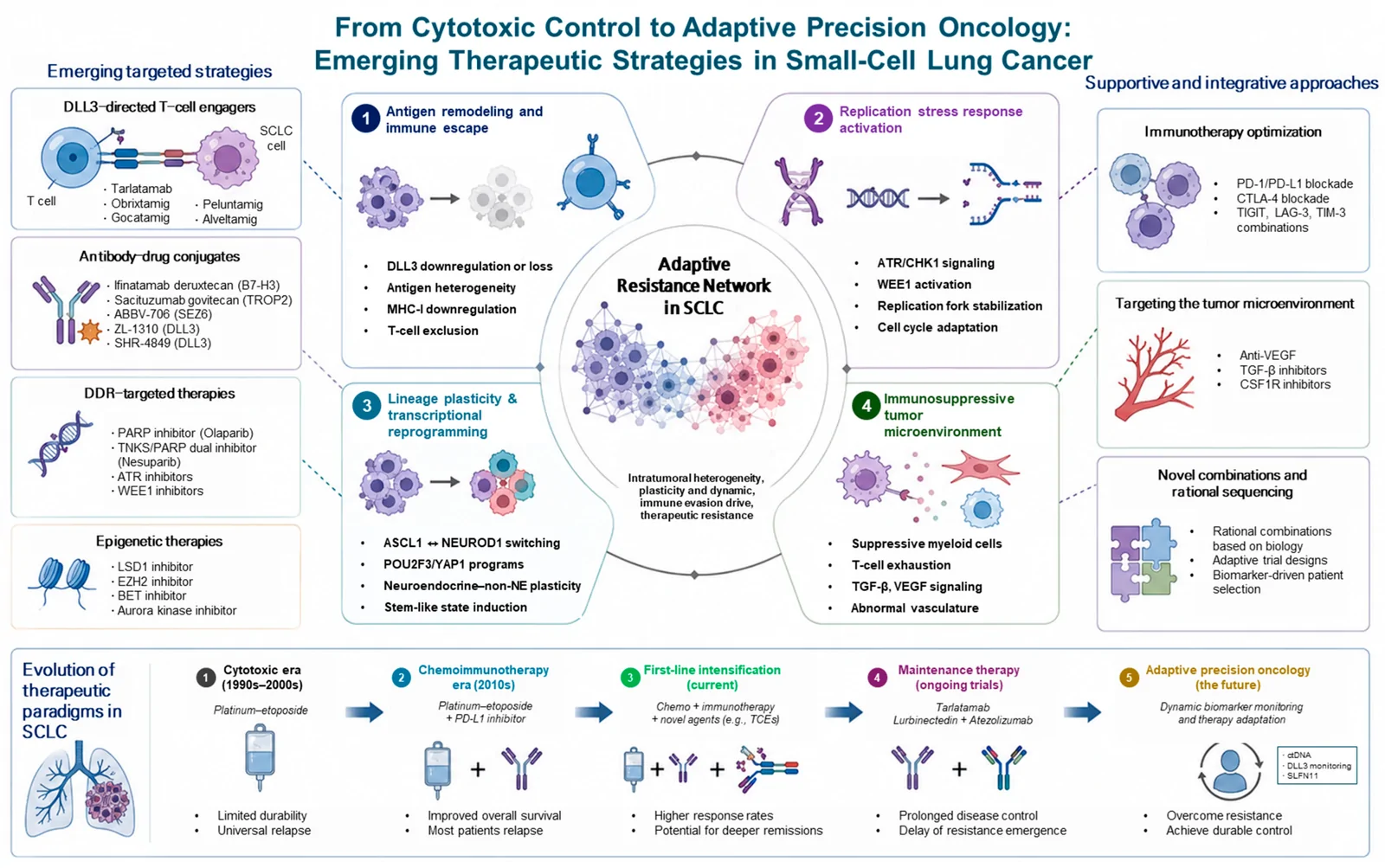
Adaptive resistance networks and evolving therapeutic strategies in SCLC. The schematic integrates four interconnected mechanisms that contribute to therapeutic failure: antigen remodeling and immune escape, replication-stress adaptation, lineage plasticity and transcriptional reprogramming, and an immunosuppressive tumor microenvironment. Emerging strategies designed to address these vulnerabilities include DLL3-directed immune redirection, antibody–drug conjugates targeting DLL3 and non-DLL3 antigens, DDR-targeted therapies, epigenetic approaches, and tumor microenvironment modulation. The lower panel summarizes the transition from platinum-based chemotherapy and chemoimmunotherapy toward first-line intensification, maintenance-based residual disease suppression, and biomarker-guided adaptive therapy. The clinical development examples shown predominantly relate to extensive-stage disease. Abbreviations: ADC, antibody–drug conjugate; DDR, DNA damage response; NE, neuroendocrine; SCLC, small-cell lung cancer; TME, tumor microenvironment.

These biological insights have accelerated the development of antigen-directed, immune-redirection, and replication-stress-targeted strategies. Delta-like ligand 3 (DLL3) has emerged as the most clinically validated surface target, with tarlatamab establishing proof of concept for DLL3-directed T-cell engagement in relapsed SCLC [9,10,11]. Additional antibody–drug conjugates (ADCs), bispecific antibodies, cellular therapies, and DNA damage response (DDR)-targeting approaches are under clinical investigation to address antigen heterogeneity, immune escape, and replication-stress adaptation [2,12,13]. Figure 1 provides an overview of the emerging therapeutic strategies discussed in this review and the adaptive resistance mechanisms that may limit their durability.

In this review, we examine how adaptive resistance mechanisms, including antigen remodeling, replication-stress adaptation, lineage plasticity, and immune suppression, influence both the development and clinical implementation of emerging therapies in SCLC. Rather than providing an exhaustive catalogue of investigational agents, we focus on clinically relevant therapeutic platforms, including antigen-directed therapies, immune-redirection strategies, DDR-targeting approaches, and biomarker-guided treatment concepts. Particular attention is given to how these strategies interact with the dynamic biological features of SCLC and how treatment pressure may alter target expression, lineage state, replication-stress dependency, and immune responsiveness over time. The clinical discussion primarily emphasizes extensive-stage SCLC, in which most contemporary evidence for these approaches has been generated and in which the need for more durable treatment strategies remains greatest. We also critically discuss major translational barriers, including subtype instability, limited tissue availability, biomarker reproducibility, intratumoral heterogeneity, antigen escape, cumulative toxicity, and therapy-induced resistance. Finally, we propose that future therapeutic development should incorporate treatment timing, longitudinal biomarker assessment, and resistance-informed sequencing rather than relying solely on baseline molecular classification or conventional line-of-therapy frameworks.

## 2. Literature Search Strategy

This narrative review was based on searches of PubMed/MEDLINE, ClinicalTrials.gov, relevant regulatory agency sources, and proceedings from major oncology meetings, including the American Society of Clinical Oncology, European Society for Medical Oncology, World Conference on Lung Cancer, and American Association for Cancer Research. The literature search was updated through 15 July 2026. Search terms included combinations of “small-cell lung cancer,” “adaptive resistance,” “molecular subtype,” “lineage plasticity,” “DLL3,” “tarlatamab,” “T-cell engager,” “antibody–drug conjugate,” “B7 homolog 3 (B7-H3),” “trophoblast cell-surface antigen 2 (TROP2),” “seizure-related 6 homolog (SEZ6),” “poly(ADP-ribose) polymerase (PARP),” “ATR (ataxia telangiectasia and Rad3-related),” “WEE1 kinase (WEE1),” “checkpoint kinase 1 (CHK1),” “lysine-specific demethylase 1 (LSD1),” “enhancer of zeste homolog 2 (EZH2),” “circulating tumor DNA (ctDNA),” and “maintenance therapy.”

Priority was given to prospective clinical trials, pivotal translational studies, regulatory documents, and recent studies directly addressing therapeutic resistance or clinical development in SCLC. Seminal older studies were included when necessary to establish biological or therapeutic context. Studies with limited relevance to SCLC, insufficient methodological detail, or substantial overlap with more recent reports were not prioritized. The clinical synthesis primarily focused on extensive-stage SCLC (ES-SCLC), whereas studies in limited-stage disease were included only when relevant to shared disease biology or cross-stage therapeutic development. Because this was a narrative review, no formal meta-analysis or systematic risk-of-bias assessment was performed.

## 3. Biological Basis of Therapeutic Resistance in SCLC

### 3.1. Replication Stress and DDR Dependency

A defining therapeutic vulnerability of SCLC is its dependence on mechanisms that buffer chronic replication stress. Rapid cell-cycle progression, oncogene-driven transcriptional amplification, and defective checkpoint control generate persistent replication-associated DNA damage, requiring continuous activity of DDR pathways for tumor-cell survival [13]. In particular, dependence on ATR–CHK1, WEE1, and PARP signaling creates potential opportunities for synthetic-lethal therapeutic strategies.

Among these pathways, PARP has received particular attention because of its role in single-strand DNA break repair and replication-fork stability. Preclinical studies demonstrated elevated PARP1 expression in SCLC compared with many other tumor types and suggested increased dependence on PARP-mediated DNA repair in neuroendocrine lung cancer models [14]. These findings initially generated considerable interest in PARP inhibition. However, early clinical trials of PARP inhibitor monotherapy yielded inconsistent efficacy, highlighting the heterogeneity of replication-stress dependency and the limitations of biomarker-unselected treatment strategies.

Subsequent translational investigations identified Schlafen family member 11 (SLFN11), a DNA/RNA helicase-associated protein involved in replication fork arrest, as a potentially important predictive biomarker for DDR-targeted therapies [15]. Tumors with elevated SLFN11 expression appear more susceptible to DNA-damaging agents and PARP inhibition because of impaired tolerance to replication-associated genomic stress. Nevertheless, biomarker reproducibility, intratumoral heterogeneity, and temporal instability of SLFN11 expression continue to complicate clinical implementation. More recently, interest has expanded beyond PARP inhibition toward targeting broader replication stress adaptation networks including ATR, WEE1, and CHK1, particularly in rational combination strategies involving chemotherapy, radiotherapy, or immunotherapy [16].

Importantly, replication stress in SCLC is increasingly recognized not merely as a passive consequence of rapid proliferation, but rather as a central biological dependency that actively shapes therapeutic vulnerability and resistance evolution. This concept has substantially influenced contemporary drug development strategies and has contributed to the growing interest in biomarker-driven DDR-targeting approaches in SCLC.

### 3.2. Molecular Subtypes, Lineage Plasticity, and Biomarker Evolution

Historically, SCLC was regarded as a relatively homogeneous neuroendocrine malignancy defined primarily by its characteristic histologic features and initial sensitivity to platinum-based chemotherapy. However, molecular and transcriptional profiling has demonstrated that SCLC comprises biologically distinct and dynamically evolving states with divergent lineage programs, immune phenotypes, and therapeutic vulnerabilities [7,8]. Current frameworks broadly classify SCLC into *ASCL1*-dominant (SCLC-A), *NEUROD1*-dominant (SCLC-N), POU2F3-dominant (SCLC-P), and inflamed (SCLC-I) states [8]. These categories represent functional biological programs rather than fixed or mutually exclusive disease entities. YAP1 expression has also been associated with selected non-neuroendocrine phenotypes, although it is not uniformly considered equivalent to the SCLC-I subtype.

SCLC-A tumors generally retain strong neuroendocrine characteristics and frequently express DLL3 and B-cell lymphoma 2 (BCL2), supporting the development of DLL3-directed and apoptosis-associated strategies [7,8]. SCLC-N tumors are commonly associated with MYC proto-oncogene *(MYC)*-related transcriptional programs, high proliferative activity, and potential sensitivity to Aurora kinase inhibition [17,18]. SCLC-P tumors exhibit tuft-cell-like features and reduced expression of canonical neuroendocrine markers, whereas SCLC-I tumors demonstrate relatively greater immune infiltration, antigen-presentation activity, and stromal interaction [7,8].

**Figure 2:**
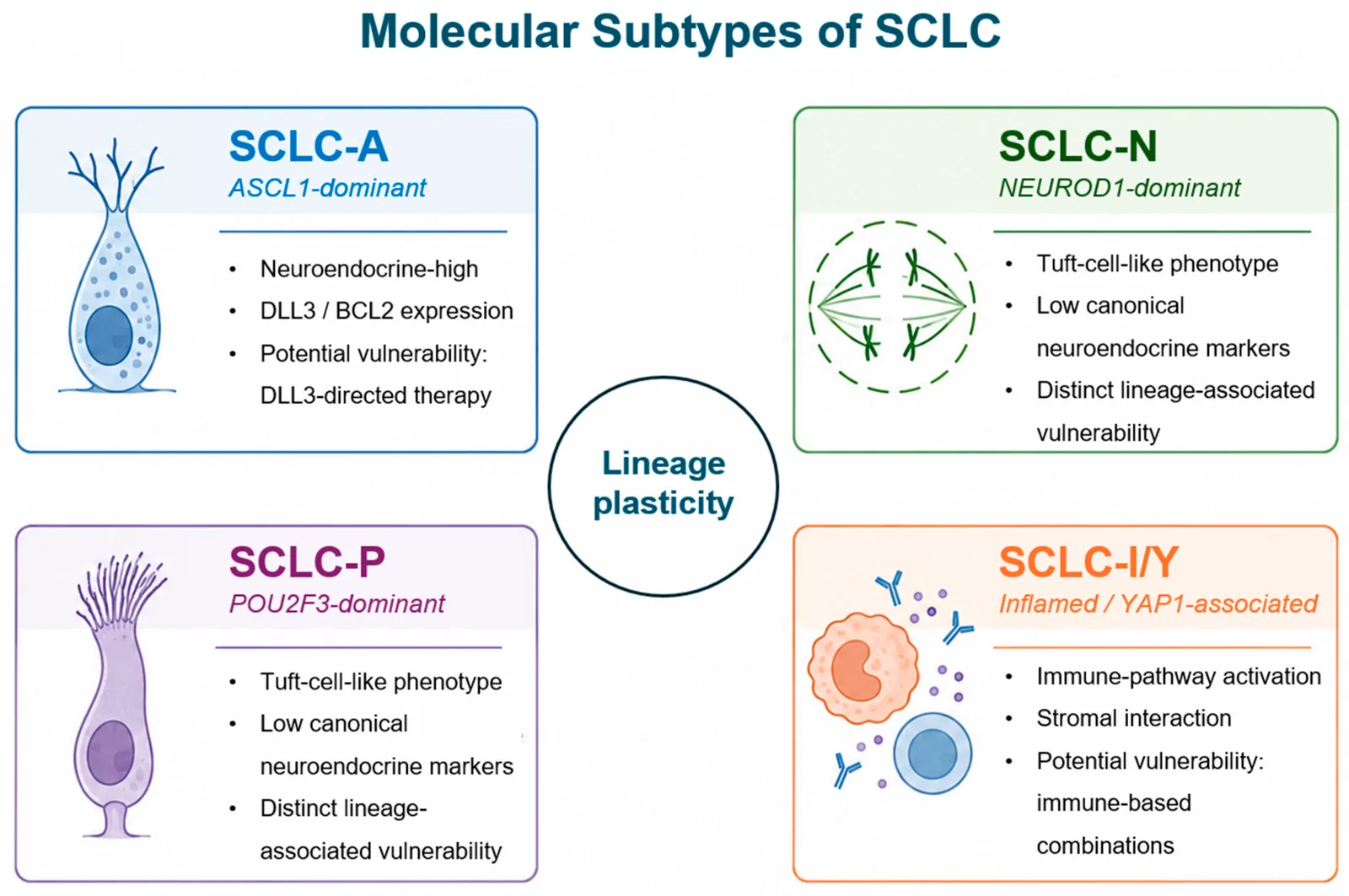
Molecular subtypes and lineage plasticity in SCLC. SCLC is broadly classified into *ASCL1*-dominant (SCLC-A), *NEUROD1*-dominant (SCLC-N), *POU2F3*-dominant (SCLC-P), and inflamed (SCLC-I) states, each associated with distinct biological features and potential therapeutic vulnerabilities. YAP1-associated non-neuroendocrine features may overlap with, but are not equivalent to, SCLC-I. Bidirectional arrows indicate lineage plasticity and subtype transition.

These subtype states are not biologically static. Therapeutic pressure, epigenetic remodeling, and microenvironmental adaptation can induce lineage transitions that alter antigen expression, immune phenotype, replication-stress dependency, and apoptotic susceptibility [12,18]. For example, loss of neuroendocrine differentiation may reduce DLL3 expression and thereby diminish the activity of DLL3-directed therapies [9]. Accordingly, the bidirectional transitions illustrated in Figure 2 emphasize that subtype classification should be interpreted as a dynamic continuum rather than a permanent tumor identity.

DLL3 expression remains a major biomarker for antigen-directed approaches, whereas SLFN11 expression and replication-stress signatures may help identify tumors susceptible to DDR-targeted therapies [19,20]. Similarly, *MYC* amplification and *NEUROD1*-associated transcriptional states may indicate sensitivity to Aurora kinase inhibition, while *ASCL1*-driven tumors may exhibit dependence on BCL2 and other neuroendocrine-associated pathways [17,18]. Nevertheless, limited tissue availability, intratumoral heterogeneity, and temporal biomarker instability restrict the reliability of static baseline assessments. Serial tissue sampling, ctDNA profiling, longitudinal transcriptional monitoring, and integrated multi-omic approaches are therefore being explored to capture subtype evolution and support adaptive therapeutic selection [7,8,18]. Collectively, molecular heterogeneity and lineage plasticity represent central determinants of therapeutic resistance in SCLC and provide a rationale for dynamic, subtype-informed biomarker strategies.

### 3.3. Immunologically Suppressive Tumor Microenvironment

Despite its exceptionally high tumor mutational burden, SCLC has historically demonstrated relatively modest responsiveness to immune checkpoint inhibitors compared with several non-small-cell lung cancer subtypes. This apparent paradox reflects the profoundly immunosuppressive tumor microenvironment characteristic of SCLC and underscores the limitations of relying solely on mutational burden as a predictor of immunotherapy sensitivity.

SCLC tumors frequently exhibit reduced major histocompatibility complex class I expression, impaired antigen presentation machinery, limited dendritic cell activation, and sparse infiltration of functional cytotoxic T lymphocytes [12]. In addition, SCLC commonly develops highly suppressive myeloid-rich microenvironments characterized by tumor-associated macrophages, myeloid-derived suppressor cells, and inhibitory cytokine signaling, collectively contributing to immune evasion and T-cell dysfunction.

Neuroendocrine differentiation itself may also contribute directly to immune suppression. Highly neuroendocrine SCLC states are often associated with diminished interferon signaling, reduced antigen presentation, and limited inflammatory pathway activation. Conversely, inflamed or YAP1-associated subtypes may demonstrate relatively enhanced immune infiltration and increased expression of immune-related genes, potentially contributing to differential responsiveness to immunotherapeutic approaches [8].

These immunologic characteristics have important therapeutic implications. While PD-L1 blockade combined with platinum–etoposide modestly improved survival in ES-SCLC, the absolute magnitude of benefit remains limited, and durable long-term responders remain relatively uncommon [4,5]. Consequently, contemporary therapeutic development increasingly seeks to overcome endogenous immune resistance through active immune-redirection strategies rather than relying exclusively on restoration of pre-existing antitumor immunity.

These immunologic features provide a rationale for active immune-redirection strategies. Unlike conventional immune checkpoint inhibitors, T-cell engagers and related antigen-directed platforms recruit cytotoxic immune effector cells independently of endogenous T-cell priming and may therefore help overcome the immune-excluded microenvironment of SCLC. The immunosuppressive tumor microenvironment thus represents both a major resistance mechanism and a therapeutic target.

## 4. Surface Antigen-Directed Therapeutic Strategies

### 4.1. DLL3-Targeted Therapeutic Strategies

DLL3-directed therapy represents the most clinically established antigen-targeted strategy in SCLC. DLL3 is an inhibitory Notch ligand frequently expressed on neuroendocrine SCLC cells, particularly in *ASCL1*-dominant tumors, while showing limited expression in normal adult tissues [9]. DLL3 expression has been reported in approximately 70–85% of SCLC tumors, supporting its development as a tumor-associated therapeutic target [21].

Initial DLL3-directed development focused on rovalpituzumab tesirine (Rova-T), an ADC comprising a DLL3-targeting antibody linked to a pyrrolobenzodiazepine payload. Although early studies suggested antitumor activity in DLL3-high relapsed SCLC, the phase III TAHOE and MERU trials failed to demonstrate a survival benefit and were associated with substantial toxicities, including pleural effusion, thrombocytopenia, edema, and skin-related adverse events [10,21]. These findings did not invalidate DLL3 as a therapeutic target but highlighted limitations of the first-generation platform, including payload-related toxicity, a narrow therapeutic index, heterogeneous antigen expression, and limited durability of direct cytotoxic targeting [22].

Subsequent development shifted toward DLL3-directed immune-redirection strategies, particularly T-cell engagers (TCEs) that recruit cytotoxic T cells to DLL3-expressing tumor cells [23]. Compared with ADCs, TCEs provide a distinct mechanism based on direct immune-cell recruitment and serial tumor-cell killing. This approach may remain active when endogenous T-cell priming is limited, although efficacy may still be affected by antigen density, T-cell fitness, and the immunosuppressive tumor microenvironment [2,12].

Tarlatamab is a DLL3/CD3 bispecific TCE that induces direct T-cell-mediated cytotoxicity independently of conventional antigen presentation. In the phase II DeLLphi-301 study, tarlatamab achieved an objective response rate of approximately 40%, with responses lasting more than 9 months in previously treated SCLC [23]. Activity was also observed after prior immune checkpoint inhibitor therapy, suggesting that direct immune redirection may remain effective despite resistance to checkpoint blockade.

The clinical use of tarlatamab requires step-up dosing and structured monitoring for cytokine release syndrome (CRS) and neurologic toxicity, including immune effector cell-associated neurotoxicity syndrome (ICANS). These events occur predominantly during the initial treatment period and are generally manageable with appropriate monitoring and supportive care [23]. Patient selection should therefore consider performance status, baseline neurologic symptoms, disease tempo, comorbidities, caregiver support, and access to a treatment center experienced in managing TCE-related toxicities. Important uncertainties remain regarding treatment sequencing, use in frail patients or those with symptomatic central nervous system disease, and the predictive value of baseline or dynamic DLL3 expression. Earlier-line and maintenance applications are under investigation but remain investigational.

Additional DLL3-directed platforms include next-generation ADCs, multispecific antibodies, natural killer cell engagers, and chimeric antigen receptor T-cell therapies [2,24,25]. Among cellular immunotherapy approaches, DLL3-directed chimeric antigen receptor T cell (CAR-T) therapies represent an emerging strategy designed to provide sustained antigen-specific immune surveillance beyond antibody-based modalities. AMG 119 is the first DLL3-directed CAR-T therapy to enter clinical evaluation in relapsed SCLC, although its development remains at an early stage owing to challenges including manufacturing complexity, cytokine release syndrome, limited trafficking into bulky solid tumors, and intratumoral antigen heterogeneity [26,27]. In parallel, DLL3-directed chimeric antigen receptor natural killer cell (CAR-NK) platforms have been developed as a potential off-the-shelf alternative that may offer reduced manufacturing time, broader availability, and a potentially lower risk of severe immune-related toxicities while retaining potent cytotoxic activity against DLL3-expressing tumor cells. Although current evidence is largely limited to preclinical studies, DLL3-directed CAR-NK cells have demonstrated promising antitumor activity in SCLC models, supporting further clinical translation [28]. However, DLL3-directed treatment remains susceptible to antigen heterogeneity, lineage plasticity, and immune dysfunction. Neuroendocrine-to-non-neuroendocrine transition may reduce DLL3 expression and promote antigen escape, whereas prolonged T-cell engagement may contribute to T-cell exhaustion and adaptive immune suppression [8,12,18]. These limitations support longitudinal biomarker assessment and rational combination strategies aimed at improving the durability of DLL3-directed therapy.

The transition from Rova-T to T-cell-engaging and cellular platforms indicates that the clinical value of DLL3 targeting depends strongly on therapeutic modality, tolerability, and the capacity to overcome antigen and immune escape.

### 4.2. Emerging ADC Targets Beyond DLL3

Increasing recognition of intratumoral heterogeneity, lineage plasticity, and treatment-induced antigen remodeling has encouraged the development of ADCs targeting surface antigens beyond DLL3. Such diversification may broaden the applicability of antigen-directed treatment and reduce dependence on a single, dynamically regulated target.

B7-H3 is frequently expressed in SCLC and other neuroendocrine malignancies and has been associated with immune suppression, tumor progression, and poor clinical outcomes [29,30]. Early clinical activity with B7-H3-directed ADCs, including ifinatamab deruxtecan and risvutatug rezetecan, has supported further development of this target in SCLC [31,32]. TROP2, which is expressed across a broader range of epithelial phenotypes than DLL3, may extend antibody–drug conjugate (ADC)-based treatment beyond neuroendocrine-dominant tumors and is currently being evaluated clinically with sacituzumab govitecan in relapsed ES-SCLC [27,33]. By contrast, SEZ6 is preferentially enriched in neuroendocrine malignancies while showing limited expression in most normal tissues, providing a potential basis for more tumor-selective targeting with emerging ADCs such as ABBV-706 [34,35].

Next-generation DLL3-directed ADCs are also being developed to address limitations encountered with Rova-T. Agents such as ZL-1310 incorporate alternative payloads, linker technologies, and bystander effects intended to improve antitumor activity and tolerability [24,27]. Although these agents remain investigational, their development suggests that the earlier failure of Rova-T may have reflected platform-specific limitations rather than invalidation of DLL3 as an ADC target.

Overall, B7-H3, TROP2, SEZ6, and next-generation DLL3 platforms may support complementary rather than mutually exclusive treatment strategies. Their clinical utility will depend on the reproducibility of target-expression assays, intratumoral antigen heterogeneity, payload-related toxicity, and the ability to define biomarker-enriched populations. Longitudinal assessment of antigen expression may therefore be necessary to guide ADC selection and sequencing in SCLC.

### 4.3. Bispecific Antibodies and Immune-Redirection Strategies

Immune-redirection strategies actively recruit cytotoxic immune effector cells to tumor-associated antigens and therefore differ mechanistically from immune checkpoint inhibitors, which primarily restore pre-existing antitumor immunity. This distinction may be relevant in SCLC, where reduced antigen presentation, limited cytotoxic T-cell infiltration, impaired interferon signaling, and myeloid-mediated immune suppression frequently restrict endogenous immune responses [12,36].

The clinical activity of tarlatamab has established proof of concept for DLL3-directed T-cell engagement in previously treated SCLC, as discussed in Section 4.1. Several additional DLL3-directed platforms are now under investigation, including obrixtamig (BI 764532), gocatamig (HPN328), peluntamig (PT217) and alveltamig (ZG006) [37,38,39,40]. These agents employ bispecific, trispecific, or multispecific formats intended to enhance immune-cell recruitment, prolong target engagement, or improve activity across variable levels of DLL3 expression. However, whether these platforms provide clinically meaningful advantages over established DLL3-directed therapy remains to be determined.

Other bispecific approaches seek to modulate the tumor microenvironment rather than directly engage a tumor antigen. Dual inhibition of vascular endothelial growth factor (VEGF) and PD-1 or PD-L1 may simultaneously reduce angiogenesis and alleviate VEGF-associated immune suppression. VEGF signaling can impair dendritic-cell function, restrict cytotoxic T-cell activity, and promote recruitment of immunosuppressive myeloid populations, providing a biological rationale for combined angiogenic and immune blockade in SCLC [41,42]. Nevertheless, the relative contribution of each component and the optimal clinical setting for these agents require confirmation in randomized studies.

The durability of antigen-directed therapies may be limited by CRS, ICANS, T-cell exhaustion, antigen loss, and lineage plasticity [12,18,23]. Treatment-related cytopenias and overlapping toxicities may also complicate combinations with chemotherapy, ADCs, or DDR-targeting agents. Future development should therefore prioritize comparative clinical evidence, rational sequencing strategies, and biomarkers capable of longitudinally monitoring antigen expression and immune function.

Comparative clinical evidence, toxicity management, and longitudinal biomarker assessment will ultimately determine the relative roles and sequencing of these antigen-directed platforms, which are summarized in Table 1.

## 5. DNA Damage Response and Replication-Stress-Targeting Strategies

### 5.1. PARP Inhibition, Biomarker Selection, and Adaptive Replication-Stress Responses

SCLC exhibits extensive genomic instability, frequent inactivation of the tumor suppressor genes *TP53* and *RB1*, and high proliferative activity, resulting in sustained replication-associated stress [1,2]. These features increase dependence on DDR and cell-cycle checkpoint pathways that permit continued proliferation despite ongoing genomic damage. Proteomic and transcriptional studies have accordingly identified elevated expression or activity of replication-stress-associated proteins, including PARP1, ATR, CHK1, and WEE1, in subsets of SCLC [14,43,44].

PARP inhibition was initially pursued because of elevated PARP1 expression and preclinical sensitivity in SCLC models. By impairing single-strand DNA break repair and promoting replication-fork collapse, PARP inhibitors may increase the cytotoxic effects of endogenous replication stress and DNA-damaging treatment. Preclinical studies demonstrated enhanced antitumor activity when PARP inhibitors were combined with temozolomide, platinum-based chemotherapy, or radiotherapy, supporting subsequent clinical evaluation of veliparib, olaparib, talazoparib, and niraparib [45,46].

Clinical outcomes, however, have been less consistent than anticipated from preclinical models. In a randomized phase II study of temozolomide with or without veliparib in relapsed SCLC, veliparib improved the objective response rate and progression-free survival but did not significantly prolong overall survival in the overall study population [15,46]. These findings indicate that replication-stress dependency alone may not be sufficient for patient selection and that PARP inhibition may require biomarker enrichment or rational combination strategies to produce durable clinical benefit.

The identification of SLFN11 as a candidate predictive biomarker has provided a potential framework for improving patient selection. SLFN11 limits replication in response to DNA damage and has been associated with increased sensitivity to PARP inhibitors and cytotoxic chemotherapy in SCLC models [19,47,48]. Nevertheless, the clinical utility of SLFN11 remains limited by intratumoral heterogeneity, assay variability, epigenetic silencing, and temporal changes during treatment. These limitations emphasize that a single baseline measurement may not adequately reflect replication-stress vulnerability throughout disease progression.

Resistance to PARP inhibition may also arise through compensatory activation of ATR, CHK1, and WEE1 signaling, stabilization of replication forks, and adaptive transcriptional reprogramming [44]. Consequently, current development has increasingly shifted toward combinations that inhibit multiple components of the replication-stress response or integrate DDR-targeted therapy with chemotherapy, radiotherapy, or immunotherapy. Although these strategies may increase antitumor activity, overlapping hematologic toxicity and limited therapeutic windows remain important barriers to clinical implementation.

Multi-targeted agents may provide another approach to addressing adaptive resistance. Nesuparib (JPI-547), a dual tankyrase 1/2 and PARP1/2 inhibitor, demonstrated preclinical activity in *BRCA*-wild-type SCLC models, including growth inhibition in NCI-H146 xenografts and enhanced activity in combination with irinotecan [49]. In addition to PARP inhibition, nesuparib modulated Wnt and Hippo signaling, suggesting a potential connection between replication-stress targeting and pathways involved in lineage plasticity and tumor-cell persistence. However, these findings remain preclinical, and further studies are required to determine whether simultaneous PARP and tankyrase inhibition provides clinically meaningful advantages over established PARP-directed strategies.

The experience with PARP inhibitors indicates that DDR dependency is heterogeneous and that PARP-directed therapy is unlikely to be uniformly effective without biomarker-enriched patient selection or carefully designed combination strategies.

### 5.2. ATR, WEE1, and CHK1 Inhibition: Targeting Checkpoint Dependence

The limited and heterogeneous activity of PARP inhibitors in SCLC has encouraged investigation of additional components of the replication-stress response, particularly ATR, WEE1, and CHK1. These checkpoint kinases enable tumor cells to stabilize stalled replication forks, delay cell-cycle progression, and repair DNA damage before mitotic entry. Their inhibition may therefore increase the vulnerability of SCLC cells to endogenous replication stress and DNA-damaging therapies [50,51,52,53,54].

ATR functions as a central sensor of replication-associated DNA damage and activates CHK1 in response to stalled replication forks. Because SCLC frequently exhibits rapid proliferation and defective cell-cycle regulation, ATR inhibition may promote replication-fork collapse and mitotic failure. Preclinical studies have demonstrated enhanced antitumor activity when ATR inhibitors were combined with platinum agents, topoisomerase inhibitors, or PARP inhibitors in SCLC models [50]. Clinically, the ATR inhibitor ceralasertib has shown encouraging activity in a biomarker-driven phase II umbrella trial in combination with olaparib, supporting ATR blockade as a promising strategy for biomarker-selected SCLC, particularly in tumors characterized by high replication stress and SLFN11 expression. The CHK1 inhibitor prexasertib has demonstrated robust preclinical activity and early clinical promise by disrupting the S-phase checkpoint, although patient selection and predictive biomarkers remain to be established [55]. However, the overlapping functions of ATR and CHK1 suggest that their therapeutic value may depend more on rational combination design than on isolated pathway inhibition.

WEE1 represents a related vulnerability because it restrains CDK1 activity and prevents premature entry into mitosis. SCLC tumors with loss of functional *TP53* frequently lack an effective G1/S checkpoint and may therefore rely more heavily on WEE1-mediated G2/M arrest following DNA damage [52]. WEE1 inhibition can force cells with unresolved genomic lesions into mitosis, resulting in mitotic catastrophe. Preclinical studies have supported combinations of WEE1 inhibitors with chemotherapy, PARP inhibition, and other replication-stress-targeting agents, although the extent to which *TP53* status alone predicts clinical sensitivity remains uncertain. Among WEE1 inhibitors, adavosertib has demonstrated the feasibility of combination treatment with olaparib in early-phase clinical studies of refractory solid tumors, supporting continued evaluation of WEE1-directed strategies in replication-stress-driven malignancies, including SCLC [56].

Clinical translation of ATR-, WEE1-, and CHK1-directed strategies remains challenging. These pathways are also required for the survival of normal proliferating tissues, resulting in a relatively narrow therapeutic window. Myelosuppression, gastrointestinal toxicity, and fatigue may limit dose intensity, particularly when checkpoint inhibitors are combined with chemotherapy or radiotherapy. Consequently, treatment schedule, dose sequencing, and recovery intervals may be as important as the selection of individual agents.

Resistance may also emerge through restoration of replication-fork stability, compensatory checkpoint signaling, epigenetic reprogramming, or transition toward less replication-stress-dependent cellular states [18]. These adaptive mechanisms further limit the rationale for empiric use in unselected patients.

### 5.3. Mechanism-Based Combination Strategies and Translational Barriers

The functional redundancy of DDR pathways in SCLC provides a rationale for mechanism-based combination strategies rather than uniform use of single-agent DDR inhibitors. Such combinations aim to increase replication-associated damage while simultaneously limiting compensatory checkpoint activation and repair. However, their clinical feasibility depends on achieving sufficient pathway suppression without unacceptable toxicity.

Combining DDR inhibitors with DNA-damaging treatment represents the most direct approach. Platinum agents, topoisomerase inhibitors, alkylating agents, and radiotherapy increase DNA lesions and replication stress, potentially enhancing sensitivity to PARP, ATR, or WEE1 inhibition [50,51,52,53,54,55,56]. Nevertheless, overlapping myelosuppression and gastrointestinal toxicity may restrict dose intensity and treatment duration. The sequence and timing of each agent may therefore be critical, as intermittent or sequential administration could preserve antitumor activity while allowing recovery of normal proliferating tissues.

DDR-targeted therapy may also interact with antitumor immunity. Accumulation of cytosolic DNA can activate the cyclic GMP–AMP synthase–stimulator of interferon genes (cGAS–STING) pathway, promote inflammatory signaling, and potentially enhance immune recognition [57]. This mechanism supports combinations with immune checkpoint inhibitors or immune-redirection platforms. However, increased DNA damage does not uniformly translate into effective antitumor immunity, particularly in SCLC tumors with impaired antigen presentation, T-cell exclusion, or myeloid-mediated suppression. Clinical development should therefore evaluate both tumor-intrinsic DDR activity and the immune context in which these combinations are administered.

Epigenetic regulators provide another potential combination strategy because lineage state and replication-stress tolerance are closely interconnected. LSD1- and EZH2-directed therapies may alter neuroendocrine differentiation, chromatin accessibility, and checkpoint dependency, thereby modifying sensitivity to DDR inhibition [58]. Although this approach could theoretically restrict adaptive lineage transition while increasing replication stress, current evidence remains predominantly preclinical, and the optimal therapeutic sequence has not been established.

Several translational barriers remain. SLFN11 protein expression is among the most extensively studied candidate biomarkers, but limited tissue availability, intratumoral heterogeneity, assay variability, epigenetic silencing, and treatment-induced changes complicate its clinical use [19]. Lineage plasticity may further alter replication-stress dependency, immune phenotype, and apoptotic susceptibility during treatment [18]. These observations indicate that resistance to DDR-targeted therapy is not determined by a single repair pathway but emerges from dynamic interactions among checkpoint signaling, transcriptional reprogramming, lineage evolution, and the tumor microenvironment.

Clinical translation will therefore require biomarker-enriched populations, pharmacodynamically informed dosing schedules, and serial assessment of replication-stress and lineage states. The value of DDR-directed therapy will depend on whether these vulnerabilities can be safely targeted and reassessed as the biological state of SCLC evolves. Representative strategies and translational challenges are summarized in Table 2.

## 6. Epigenetic and Transcriptional Dependency Targeting

### 6.1. LSD1 and EZH2: Epigenetic Regulation of Lineage State and DDR Sensitivity

SCLC is characterized not only by genomic instability but also by substantial epigenetic and transcriptional plasticity. Unlike tumors in which stable genomic alterations define persistent therapeutic dependencies, SCLC can transition between lineage-associated states during disease progression and treatment [7,18]. These transitions may alter surface-antigen expression, immune phenotype, replication-stress dependence, and apoptotic susceptibility. Epigenetic regulators should therefore be considered not simply as independent drug targets but as determinants of the therapeutic state of the tumor.

LSD1, encoded by *KDM1A*, contributes to the maintenance of neuroendocrine transcriptional programs and repression of NOTCH-associated lineage differentiation. This function appears particularly relevant in *ASCL1*-dominant neuroendocrine SCLC, in which LSD1 supports lineage identity through chromatin-dependent transcriptional regulation [58]. Preclinical studies have shown that LSD1 inhibition can activate NOTCH signaling, reduce neuroendocrine marker expression, and suppress tumor growth [59].

However, alteration of lineage identity may have context-dependent consequences. Loss of neuroendocrine differentiation may impair the growth of selected SCLC populations but may also promote transition toward alternative cellular states that are less dependent on the original therapeutic vulnerability [18]. Moreover, because DLL3 expression is closely associated with neuroendocrine lineage programs, LSD1-induced lineage reprogramming could theoretically reduce susceptibility to DLL3-directed treatment. The clinical value of LSD1 inhibition may therefore depend on treatment sequence, the extent of lineage conversion, and whether the resulting state creates a new actionable vulnerability rather than simply facilitating therapeutic escape.

EZH2, the catalytic component of polycomb repressive complex 2, represents another link between epigenetic regulation and treatment resistance. Through histone H3 lysine 27 trimethylation, EZH2 regulates chromatin accessibility, lineage-state stability, and transcriptional repression [60,61]. Of particular relevance to DDR-targeted therapy, EZH2-mediated silencing of *SLFN11* has been associated with reduced sensitivity to chemotherapy and PARP inhibition. In preclinical models, EZH2 inhibition restored SLFN11 expression and partially resensitized resistant tumors to DNA-damaging treatment [19].

This EZH2–SLFN11 relationship provides a mechanistic bridge between chromatin remodeling and replication-stress adaptation. Nevertheless, restoration of SLFN11 expression has not yet been established as a clinically validated strategy for reversing DDR resistance. EZH2 may also influence antigen presentation and inflammatory signaling, but whether epigenetic modulation can reproducibly enhance immune responsiveness in SCLC remains uncertain. Future studies should therefore determine whether LSD1 or EZH2 inhibition produces a therapeutically favorable lineage transition and identify the treatment context in which epigenetic reprogramming can be combined safely with DDR-targeted or antigen-directed therapies.

### 6.2. MYC/Aurora Kinase and Broader Transcriptional Dependencies

Activation or amplification of *MYC* is associated with increased proliferative activity, mitotic stress, reduced neuroendocrine differentiation, and transition toward *NEUROD1*-dominant or variant SCLC states [62,63,64]. These biological features provide a rationale for targeting mitotic and transcriptional dependencies that may be preferentially maintained in *MYC*-associated tumors.

Aurora kinase A regulates mitotic spindle formation and chromosomal segregation. *MYC*-driven SCLC models have demonstrated increased sensitivity to Aurora kinase inhibition, potentially because heightened proliferative and mitotic stress reduces their capacity to tolerate additional disruption of chromosome segregation [65]. Clinical evaluation of the Aurora kinase inhibitor alisertib, however, showed only modest activity in unselected patients. Exploratory analyses suggested greater benefit in *MYC*-associated tumors, but these observations require prospective validation and do not yet support routine subtype-based patient selection [17,18,62].

The discrepancy between preclinical sensitivity and limited overall clinical activity illustrates a broader challenge in targeting transcriptional dependencies. *MYC* amplification or expression alone may not adequately capture Aurora kinase dependence, and therapeutic sensitivity may additionally be influenced by lineage state, replication-stress burden, compensatory signaling, and prior treatment exposure [66]. Dynamic or composite biomarkers may therefore be more informative than a single baseline molecular alteration.

SCLC may also depend on broader transcriptional regulatory networks involving bromodomain and extraterminal (BET) proteins, enhancer-associated complexes, and chromatin-remodeling machinery. Although inhibition of these pathways has produced antitumor effects in preclinical models, early clinical development of BET inhibitors has generally shown limited single-agent durability [67]. Potential resistance mechanisms include enhancer reprogramming, activation of compensatory transcriptional pathways, and transition toward alternative lineage states.

These findings suggest that epigenetic and transcriptional agents may be more useful as biomarker-defined modulators of lineage state or treatment sensitivity than as uniform monotherapies. Prospective studies should determine whether treatment-induced reprogramming creates an actionable vulnerability or instead promotes escape from the original therapeutic target.

## 7. Therapeutic Timing and Resistance Interception in ES-SCLC

### 7.1. A Three-Window Framework for Clinical Translation

Platinum–etoposide chemotherapy combined with PD-L1 inhibition remains the standard first-line treatment for ES-SCLC. Although atezolizumab- and durvalumab-based regimens improve survival compared with chemotherapy alone, most patients eventually develop progressive disease [4,5,6]. The central clinical challenge is therefore no longer limited to increasing the initial response rate but also includes preventing the expansion of treatment-selected residual clones and delaying the emergence of biologically diversified relapse.

Emerging therapies in SCLC are commonly categorized according to drug class or molecular target. However, their clinical activity may also depend on the timing and biological context in which they are introduced. Treatment-naïve disease, post-induction residual disease, and overt relapse differ in tumor burden, clonal composition, antigen expression, immune competence, and exposure to prior selective pressure. Accordingly, we propose three clinically relevant therapeutic windows in ES-SCLC: induction, post-induction residual disease, and overt relapse. This timing-based framework complements conventional target-based classification by considering not only which therapy is administered, but also when the corresponding vulnerability is most likely to be therapeutically accessible.

This distinction is particularly relevant in SCLC because lineage-associated states are not fixed. Chemotherapy, immune checkpoint inhibition, and antigen-directed treatment may induce or select for changes in neuroendocrine differentiation, surface-antigen expression, replication-stress dependency, and immune phenotype [8,18]. An agent that shows limited activity in molecularly heterogeneous relapse may therefore remain effective during an earlier treatment window, whereas a treatment that is active after progression may introduce unnecessary toxicity or interfere with immune function when added during induction. Treatment timing should consequently be evaluated as an independent component of therapeutic development rather than being inferred solely from activity observed in another line of therapy.

### 7.2. Induction Window: Cytoreduction and Early Resistance Prevention

The induction window is characterized by high tumor burden, rapid disease tempo, and an immediate need for cytoreduction. Platinum–etoposide therefore remains an essential backbone, whereas emerging combinations seek to increase the depth of tumor-cell elimination or prevent the early establishment of resistant residual populations. Current approaches include the addition of DLL3-directed TCEs, ADCs, and agents that simultaneously modulate angiogenic and immune-suppressive signaling.

The phase III DeLLphi-312 trial is evaluating tarlatamab in combination with carboplatin, etoposide, and durvalumab versus standard chemoimmunotherapy in treatment-naïve ES-SCLC. This study will test whether DLL3-directed immune redirection can be integrated before extensive treatment-induced antigen remodeling and lineage diversification occur. However, the biological rationale for early TCE administration must be balanced against practical concerns, including cytokine release syndrome monitoring, overlapping treatment-related toxicities, and the potential effects of cytotoxic chemotherapy on T-cell number and function [68].

Immuno-angiogenic combinations represent a mechanistically distinct strategy for delaying therapeutic resistance during the post-induction treatment window. Dual VEGF and PD-1/PD-L1 blockade may enhance antitumor immunity by normalizing tumor vasculature while reducing VEGF-mediated T-cell exclusion, myeloid cell recruitment, and other immunosuppressive mechanisms within the tumor microenvironment [69]. Although early-phase studies have reported encouraging response rates, these findings alone are insufficient to demonstrate durable resistance prevention. Ultimately, randomized clinical trials are needed to determine whether enhanced initial tumor control translates into meaningful improvements in progression-free and overall survival without increasing toxicity or compromising subsequent treatment options [70].

Evaluation of first-line intensification should therefore extend beyond conventional objective response rate and progression-free survival. Relevant translational end points include treatment discontinuation, maintenance of chemotherapy dose intensity, recovery of immune-cell competence, ctDNA clearance, and the molecular characteristics of disease persisting after induction. These measurements may clarify whether an intensified regimen merely produces deeper initial cytoreduction or meaningfully alters the composition and subsequent behavior of residual disease.

### 7.3. Post-Induction Residual Disease as a Resistance-Interception Window

Post-induction residual disease should be regarded as a biologically distinct therapeutic window rather than simply a lower-volume extension of treatment-naïve disease. Patients entering maintenance therapy have already been selected for treatment sensitivity or, at minimum, disease control during induction. At the same time, the residual tumor population has been subjected to substantial therapeutic pressure and may comprise clones undergoing lineage reprogramming, antigen remodeling, adaptation to replication stress, and immune escape.

The phase III IMforte trial provides the first randomized evidence that active intervention during this window can improve clinical outcomes. Among patients without disease progression following induction with carboplatin, etoposide, and atezolizumab, maintenance lurbinectedin plus atezolizumab significantly improved median progression-free survival (5.4 vs. 2.1 months) and median overall survival (13.2 vs. 10.6 months) compared with atezolizumab alone. These findings support the concept that targeting residual disease after induction can provide clinical benefit beyond continuation of immune checkpoint blockade alone [71].

DLL3-directed maintenance is based on a complementary therapeutic rationale. In the non-randomized phase Ib DeLLphi-303 study, patients without disease progression after four to six cycles of first-line chemoimmunotherapy received tarlatamab together with continued PD-L1 inhibition. The study demonstrated the feasibility of sustained DLL3-directed immune engagement during maintenance and reported encouraging disease control. However, these findings should be interpreted cautiously because the study enrolled a highly selected population with Eastern Cooperative Oncology Group performance status 0–1, excluded patients with early progression, and lacked a randomized comparator [72].

The ongoing phase III DeLLphi-305 trial has therefore been designed to determine whether the addition of tarlatamab to maintenance durvalumab following platinum–etoposide and durvalumab induction translates into superior clinical outcomes compared with durvalumab alone [73].

The therapeutic value of the maintenance window likely reflects the coexistence of reduced tumor burden and incomplete resistance evolution. Nevertheless, residual tumors are unlikely to be biologically uniform after induction, and neither preserved DLL3 expression nor intact immune competence should be assumed across all patients. Consequently, maintenance strategies should ideally be guided by post-induction biological reassessment rather than applied uniformly. Candidate biomarkers include ctDNA dynamics, residual radiographic disease burden, DLL3 and other surface-antigen expression, lineage-associated markers, and indices of T-cell fitness. Prospective studies are needed to determine whether these features can identify patients who require treatment intensification, those most likely to benefit from specific maintenance platforms, and those for whom additional therapy is unlikely to provide meaningful benefit relative to toxicity.

### 7.4. Overt Relapse: Reassessment and Modality Switching

Overt relapse represents a biologically distinct state from both treatment-naïve and post-induction residual disease. By the time radiographic progression becomes apparent, prior therapy may have selected tumor populations with altered antigen expression, lineage identity, replication-stress dependency, apoptotic susceptibility, and immune interactions. Treatment selection based solely on the original diagnostic specimen may therefore fail to reflect the current therapeutic state of the disease.

The randomized phase III DeLLphi-304 trial established DLL3-directed T-cell engagement as an effective strategy after platinum-based treatment. Tarlatamab improved median overall survival to 13.6 months compared with 8.3 months with investigator-selected chemotherapy and was associated with fewer grade 3 or higher adverse events. These results provide comparative evidence that direct immune redirection can remain clinically active in relapsed SCLC despite prior platinum therapy and, in most patients, prior immune checkpoint inhibition [74].

The increasing availability of multiple antigen-directed platforms nevertheless creates a sequencing challenge. Resistance to DLL3-directed therapy may arise through antigen loss or heterogeneity, impaired T-cell fitness, microenvironment-mediated immune suppression, payload resistance, or defective intracellular trafficking. Because these mechanisms may have different implications for subsequent treatment, progression should be viewed as a point for biological reassessment rather than automatic continuation of a predetermined drug sequence.

These proposed relationships remain hypothesis-generating. Systematic paired analyses before and after TCE or ADC treatment are limited, and no validated algorithm currently determines whether patients should change antigen, payload, or therapeutic modality after progression. The implications of antigen status, immune competence, and modality-specific resistance for subsequent treatment selection are discussed further in Section 8.

Longitudinal cfDNA studies have shown that plasma-derived alterations can track treatment response and, in selected patients, identify relapse before conventional imaging [75,76]. However, genomic ctDNA analysis cannot fully capture surface-antigen density, transcriptional lineage state, or T-cell competence. When clinically feasible, molecular liquid-biopsy findings should therefore be integrated with repeat tissue sampling, circulating tumor cell analysis, or other phenotypic approaches.

### 7.5. Implications for Clinical Trial Design

A treatment-window framework has three principal implications for clinical development. First, efficacy and toxicity should not be assumed to transfer directly across induction, maintenance, and relapse settings. Second, biomarker sampling should be aligned with the relevant treatment window to determine whether a therapeutic vulnerability persists after treatment exposure. Third, clinical endpoints and acceptable toxicity thresholds should reflect the distinct objectives of cytoreduction, residual-disease suppression, and control of molecularly evolved relapse. The proposed framework is summarized in Table 3.

## 8. A Resistance-Informed Adaptive Sequencing Framework

Building on the treatment-window framework described in Section 7, we propose a resistance-informed approach to therapeutic sequencing based on three components: the mechanism of the preceding therapy, the current biological state of the tumor, and the anticipated mechanism of resistance. This framework is hypothesis-generating and is not intended to represent a validated clinical treatment algorithm.

### 8.1. Evolving Therapeutic States in SCLC

Therapeutic vulnerability in SCLC can be considered across three interacting biological dimensions: the antigen state, including the expression and heterogeneity of DLL3, B7-H3, TROP2, and SEZ6; the lineage and replication-stress state, including neuroendocrine differentiation, transcriptional subtype, SLFN11 expression, MYC-associated programs, and checkpoint dependence; and the immune state, including T-cell fitness, antigen presentation, immune-cell infiltration, myeloid suppression, and angiogenic signaling.

These dimensions are interconnected and may change during treatment. Neuroendocrine-to-non-neuroendocrine transition may reduce DLL3 expression while altering replication-stress dependency and immune interactions, whereas epigenetic silencing of SLFN11 may reduce sensitivity to DNA-damaging therapy without eliminating other checkpoint dependencies [8,18,19,20]. Treatment selection based on a single baseline biomarker may therefore fail to identify the vulnerability that remains after therapeutic exposure.

### 8.2. Linking Prior Therapy to Subsequent Treatment Selection

Resistance to antigen-directed therapy may result from target loss, immune-effector dysfunction, microenvironmental suppression, impaired internalization, drug efflux, or payload resistance. These mechanisms have different implications for subsequent treatment. A change in antigen may be appropriate when target expression is lost, whereas switching payload or therapeutic modality may be more relevant when the original antigen remains detectable. For example, after progression on a DLL3-directed T-cell engager, targeting B7-H3, TROP2, or SEZ6 may be reasonable when DLL3 expression is reduced, while continued DLL3 targeting with a different modality could remain biologically plausible when antigen expression is preserved but T-cell-dependent cytotoxicity is impaired.

Resistance to DDR-targeted treatment may involve SLFN11 silencing, restoration of replication-fork stability, or compensatory activation of ATR, CHK1, and WEE1 signaling [19,20]. Similarly, progression after immune checkpoint inhibition may be associated with immune exclusion or myeloid-mediated suppression, potentially supporting direct T-cell engagement or combined VEGF/PD-(L)1 blockade. These proposed sequences remain conceptual and require prospective clinical validation.

### 8.3. Longitudinal Biomarkers and Prospective Validation

Implementation of resistance-informed sequencing will require biomarkers capable of capturing tumor evolution over time. ctDNA can provide information on molecular response, clonal dynamics, and emerging relapse, but may not fully represent transcriptional subtype, surface-antigen density, or immune-cell competence [75,76]. Repeat tissue sampling and circulating tumor cell analysis may therefore provide complementary phenotypic information when clinically feasible.

Biomarker dynamics associated with outcome should not be assumed to support treatment switching. Thresholds defining clinically meaningful antigen loss, subtype transition, molecular progression, or restoration of replication-stress dependency remain unvalidated in SCLC. Prospective trials should therefore distinguish prognostic monitoring from biomarkers that can directly select or modify treatment.

Relevant trial designs could incorporate molecular assessment at diagnosis, completion of induction therapy, and progression; biomarker-stratified maintenance cohorts; or platform protocols permitting treatment reassignment after documented changes in antigen, lineage, replication-stress, or immune state. Such studies are needed to determine whether resistance-informed sequencing improves outcomes compared with conventional treatment selection based predominantly on line of therapy.

This framework shifts the emphasis from identifying a universally optimal sequence toward determining which vulnerability remains actionable after each therapeutic pressure. Representative ongoing and recently completed clinical programs are summarized in Table 4.

## 9. Conclusions

Recent advances in DLL3-directed T-cell engagement, next-generation ADCs, DDR targeting, and epigenetic modulation have expanded the therapeutic landscape of SCLC, but durable benefit remains constrained by antigen heterogeneity, lineage plasticity, replication-stress adaptation, immune dysfunction, and treatment-induced clonal selection. These limitations support a treatment strategy that considers both the timing of therapy and the biological state that persists after prior treatment. The timing- and resistance-informed framework proposed in this review remains hypothesis-generating and requires prospective validation through longitudinal molecular and phenotypic biomarker assessment. Future progress may therefore depend not only on identifying more active therapies, but also on determining when and in which evolving tumor state they should be administered.

## Figures and Tables

**Table 1:** Evolution of DLL3-Directed Therapeutic Platforms.

Platform	Representative Agent	Significance	Major Limitation	Ref.
First-generation ADC	Rova-T	First clinical validation of DLL3 targeting	Phase III failure; severe toxicity	[10]
Approved DLL3/CD3 T-cell engager	Tarlatamab	First clinically validated DLL3 immune-redirection therapy; FDA approved	CRS, ICANS, antigen escape, T-cell exhaustion	[23]
Next-generation DLL3 ADC	ZL-1310	Improved therapeutic index	Early clinical development; optimal sequencing unknown	[24]
CAR-T-cell therapy	AMG 119	Potential durable cellular therapy	Manufacturing challenges; CRS/ICANS	[26]
DLL3-directed CAR-NK platform	DLL3-CAR-NK cells	Off-the-shelf cellular therapy potential	Limited persistence; preclinical evidence	[28]
Bispecific T-cell engager	Obrixtamig (BI 764532)	Alternative T-cell engagement platform	Early clinical development; CRS; DLL3 dependence	[37]
Trispecific T-cell engager	Gocatamig (HPN328); alveltamig (ZG006)	Enhanced target avidity and exposure	Limited clinical evidence; molecular complexity	[38,39]
Innate immune bispecific antibody	Peluntamig (PT217)	T-cell-independent immune redirection	Limited clinical validation	[40]

Abbreviations: ADC, antibody–drug conjugate; CAR, chimeric antigen receptor; CRS, cytokine release syndrome; ICANS, immune effector cell-associated neurotoxicity syndrome.

**Table 2:** Replication stress-targeting strategies in SCLC.

Target	Representative Agent(s)	Biological Dependency	Clinical Rationale	Major Challenge	Potential Biomarker	Ref.
PARP	Olaparib, Veliparib, Talazoparib	Replication stress	Combination therapy	Limited durability	SLFN11	[15,19,46,47,48]
Dual TNKS/PARP	Nesuparib	Wnt/Hippo + DDR	Multi-pathway inhibition	Early development	AXIN1, Wnt/Hippo activity (exploratory)	[49]
ATR	Ceralasertib	Replication fork stress	PARP/ platinum combination	Toxicity	Replication-stress signature	[54]
CHK1	Prexasertib	S-phase checkpoint	DDR combination	Biomarker uncertainty	Replication-stress signature	[55]
WEE1	Adavosertib	G2/M checkpoint	Mitotic catastrophe	Scheduling	TP53 loss (exploratory)	[56]

Abbreviations: ATR, ataxia telangiectasia and Rad3-related; CHK1, checkpoint kinase 1; DDR, DNA damage response; PARP, poly(ADP-ribose) polymerase; TNKS, tankyrase; WEE1, WEE1 kinase.

**Table 3:** Proposed treatment-window framework for emerging therapeutic strategies in ES-SCLC.

Treatment Window	Biological Objective	Representative Strategy *	Current Evidence	Major Translational Challenge
Induction	Maximize cytoreduction while preserving immune competence	DLL3-directed T-cell engagers with chemoimmunotherapy	Phase III evaluation	CRS, antigen escape, optimal integration
	Improve targeted drug delivery	B7-H3- and TROP2-directed ADCs	Early clinical development	Toxicity, biomarker selection
Post-induction residual disease (maintenance)	Suppress residual disease	DLL3-directed and transcription-targeted maintenance strategies	Phase III evaluation	Long-term tolerability, resistance evolution
	Match therapy to the evolving tumor biology	Biomarker-guided sequencing of antigen-directed and DDR- targeted therapies	Conceptual framework	Antigen loss, lineage plasticity, limited biomarkers
	Maximize cytoreduction while preserving immune competence	DLL3-directed T-cell engagers with chemoimmunotherapy	Phase III evaluation	CRS, antigen escape, optimal integration
Relapse	Improve targeted drug delivery	B7-H3- and TROP2-directed ADCs	Early clinical development	Toxicity, biomarker selection

Abbreviations: ADC, antibody–drug conjugate; DDR, DNA damage response; ES-SCLC, extensive-stage small-cell lung cancer; CRS, cytokine release syndrome. * The treatment-window framework represents the authors’ conceptual synthesis based on the available clinical evidence discussed in this review.

**Table 4:** Representative late-stage clinical programs for emerging therapies in ES-SCLC.

Agent	Target/Platform	Clinical Setting	Key Clinical Implication	Current Status [Ref.]
Tarlatamab	DLL3 T-cell engager	≥2 L ES-SCLC	Established DLL3- directed immunotherapy with improved survival	FDA approved [23]
ZL-1310	DLL3 ADC	Relapsed ES-SCLC	Next-generation DLL3-targeted ADC	Early clinical development [24]
HS-20093 (Risvutatug rezetecan)	B7-H3 ADC	Relapsed ES-SCLC	Expanding the B7-H3 ADC platform	Phase III [32]
Pumitamig (PM8002)	VEGF/PD-L1 bispecific antibody	1 L ES-SCLC	Investigating dual angiogenic and immune checkpoint blockade	Phase III [42]
Tarlatamab + platinum–etoposide ± durvalumab	DLL3 T-cell engager	1 L ES-SCLC	Evaluating frontline integration with chemoimmunotherapy	Phase III [68]
Lurbinectedin + atezolizumab	Transcription inhibitor + PD-L1 inhibitor	Maintenance ES-SCLC	Extending disease control after induction therapy	Phase III [71]
Tarlatamab + durvalumab	DLL3 T-cell engager	Maintenance ES-SCLC	Evaluating maintenance after induction therapy	Phase III [73]
Ifinatamab deruxtecan (I-DXd)	B7-H3 ADC	Relapsed ES-SCLC	Validating B7-H3 as a therapeutic target	Phase III [77]
ABBV-706	SEZ6 ADC	Relapsed/ Refractory SCLC	Exploring SEZ6-directed ADC therapy	Phase II [78]
Lurbinectedin	Transcription inhibitor	≥2 L ES-SCLC	Accelerated approval based on single-arm activity despite negative confirmatory Phase III trial	FDA approved [79,80]
Sacituzumab govitecan	TROP2 ADC	Relapsed ES-SCLC	Evaluating TROP2-targeted ADC therapy	Phase II [81]

Abbreviations: ADC, antibody–drug conjugate; FDA, U.S. Food and Drug Administration; ES-SCLC, extensive-stage small-cell lung cancer; PD-L1, programmed death ligand 1. Representative clinical programs are shown and do not constitute an exhaustive list of ongoing clinical trials.

## Data Availability

No new data were created or analyzed in this study. Data sharing is not applicable to this article.

## Abbreviations

The following abbreviations are used in this manuscript:

ADC	Antibody–drug conjugate
*ASCL1*	Achaete-scute homolog 1
AURKA	Aurora kinase A
ATR	Ataxia telangiectasia and Rad3-related
BET	Bromodomain and extraterminal domain
B7-H3	B7 homolog 3
BCL2	B-cell lymphoma 2
CAR-T	Chimeric antigen receptor T cell
CAR-NK	Chimeric antigen receptor natural killer cell
CD3	Cluster of differentiation 3
cGAS-STING	Cyclic GMP-AMP synthase–stimulator of interferon genes
CHK1	Checkpoint kinase 1
CRS	Cytokine release syndrome
ctDNA	Circulating tumor DNA
DDR	DNA damage response
DLL3	Delta-like ligand 3
ES-SCLC	Extensive-stage small-cell lung cancer
EZH2	Enhancer of zeste homolog 2
ICANS	Immune effector cell-associated neurotoxicity syndrome
LSD1	Lysine-specific demethylase 1
*MYC*	*MYC* proto-oncogene
NEUROD1	Neuronal differentiation 1
PARP	Poly(ADP-ribose) polymerase
PD-L1	Programmed death ligand 1
POU2F3	POU class 2 homeobox 3
SEZ6	Seizure-related homolog 6
SCLC	Small-cell lung cancer
SLFN11	Schlafen family member 11
TCE	T-cell engager
TROP2	Trophoblast cell-surface antigen 2
VEGF	Vascular endothelial growth factor
WEE1	WEE1 kinase
YAP1	Yes-associated protein 1
